# Genetic risk factors for autoimmune hepatitis: implications for phenotypic heterogeneity and biomarkers for drug response

**DOI:** 10.1186/s40246-020-00301-4

**Published:** 2021-01-28

**Authors:** Takashi Higuchi, Shomi Oka, Hiroshi Furukawa, Shigeto Tohma, Hiroshi Yatsuhashi, Kiyoshi Migita

**Affiliations:** 1 Molecular and Genetic Epidemiology Laboratory, Faculty of Medicine, University of Tsukuba, 1-1-1 Tennodai, Tsukuba, 305-8575 Japan; 2 Department of Nephrology, Ushiku Aiwa General Hospital, 896 Shishiko-cho, Ushiku, 300-1296 Japan; 3 Department of Rheumatology, National Hospital Organization Tokyo National Hospital, 3-1-1 Takeoka, Kiyose, 204-8585 Japan; 4 Clinical Research Center for Allergy and Rheumatology, National Hospital Organization Sagamihara National Hospital, 18-1 Sakuradai, Minami-ku, Sagamihara, 252-0392 Japan; 5 Clinical Research Center, National Hospital Organization Nagasaki Medical Center, 2-1001-1 Kubara, Omura, 856-8562 Japan; 6 Department of Rheumatology, Fukushima Medical University School of Medicine, 1 Hikarigaoka, Fukushima, 960-1295 Japan

**Keywords:** Autoimmune hepatitis, Genetic risk factor, *HLA*, Single nucleotide variant, Liver cirrhosis

## Abstract

Autoimmune hepatitis (AIH) is a rare chronic progressive liver disease with autoimmune features. It mainly affects middle-aged women. AIH is occasionally complicated with liver cirrhosis that worsens the prognosis. Genetic and environmental factors are involved in the pathogenesis of AIH. Genetic studies of other diseases have been revealing of pathogenesis and drug efficacy. In this review, we summarize the genetic risk factors for AIH, including human leukocyte antigen (*HLA*) and non-*HLA* genes. A genome-wide association study (GWAS) on European AIH revealed the strongest associations to be with single nucleotide variants (SNVs) in *HLA*. Predisposing alleles for AIH were DRB1*03:01 and DRB1*04:01 in Europeans; DRB1*04:04, DRB1*04:05, and DRB1*13:01 in Latin Americans; and DRB1*04:01 and DRB1*04:05 in Japanese. Other risk SNVs in non-*HLA* genes for AIH were found by a candidate gene approach, but several SNVs were confirmed in replication studies. Some genetic factors of AIH overlapped with those of other autoimmune diseases. Larger-scale GWASs of other ethnic groups are required. The results of genetic studies might provide an explanation for the phenotypic heterogeneity of AIH and biomarkers for drug responses.

**Supplementary Information:**

The online version contains supplementary material available at 10.1186/s40246-020-00301-4.

## Introduction

Autoimmune hepatitis (AIH) is a chronic progressive autoimmune liver disease which mainly affects middle-aged women [1–6]. Among adult AIH patients, 70–90% were female and the peak age of onset was 60–80 years [7, 8]. AIH is a rare disease; its prevalence is 8–24.5 per 100 000 in European populations [7]. In Asia, its prevalence is similar [8, 9]. AIH is characterized by elevated levels of aspartate aminotransferase, alanine aminotransferase, or immunoglobulin G and the presence of interface hepatitis. The production of antinuclear antibodies in serum and anti-smooth muscle antibodies has been observed in type 1 AIH. Type 1 AIH shares features with systemic lupus erythematosus (SLE): the presence of lupus erythematosus cells and antinuclear antibodies. Thus, type 1 AIH was initially termed “lupoid hepatitis” [10–12]. AIH with liver kidney microsomal type 1 antibodies was designated type 2 AIH and was observed in European children. The diagnosis of AIH is often complicated by the presence of liver cirrhosis, and the pattern of progression in these patients is smoldering and latent. AIH patients diagnosed with liver cirrhosis are at higher risk of developing hepatocellular carcinoma [13]. The prognosis of AIH patients with cirrhosis was worse than for those without. In a Japanese study, the 15-year hepatocellular carcinoma-free survival rate was 62.2% for AIH with cirrhosis, compared with 96.6% for AIH without cirrhosis [14]. Although most AIH patients present a chronic and progressive course, some show acute presentation [15, 16], indicating that AIH is a heterogeneous syndrome. The criteria of the International Autoimmune Hepatitis Group (IAIHG) for the diagnosis of AIH were developed from clinical, laboratory (including the human leukocyte antigen [*HLA*] allele), and pathological findings [17].

Although the etiology of AIH is obscure, it is supposed that environmental and genetic factors are involved in its pathogenesis. The role of gene–environment interactions has also been predicted in autoimmune diseases [18]. Viral infections, low levels of vitamin D, altered microbiota, exposure to sex hormones, and the administering of drugs are candidate environmental factors in an individual’s predisposition to AIH. Infection by several viruses, including hepatitis C virus, cytomegalovirus, and herpes simplex virus type 1, are thought to cause AIH according to the molecular mimicry hypothesis [19]: self-antigens sharing amino acid sequence homologies with virus proteins would be the targets of autoimmune reactions. Low serum vitamin D levels are associated with severe histological findings of AIH [20]. Increased intestinal permeability, dysregulation of microbiota, and bacterial translocation have been noted in AIH patients [21]. AIH predominantly affects females, and the effects of sex hormones have been shown to be similar in other autoimmune diseases [22, 23]. Drug-induced hepatitis is classified separately from AIH. However, exposure to drugs remains a possible cause of AIH [24]. Since a risk of drug-induced liver injury has also been associated with *HLA* [25–30], similar mechanisms may be involved in the pathogenesis of AIH during unrecognized exposure to drugs.

A concordance of AIH in twins and AIH family accumulation were evident in a recent epidemiological study, and extrahepatic autoimmune diseases have been frequently observed in AIH patients [31–33], suggesting common genetic risk factors for AIH and other autoimmune diseases. Although *HLA* is the strongest genetic risk factor for AIH, other genetic factors associated with non-*HLA* genes have also been reported (Table 1). Since genetic studies have contributed in revealing the mechanisms of pathogenesis and drug efficacy for other diseases, genetic risk factors for AIH including *HLA* and non-*HLA* genes are summarized in this review.

**Table 1:** Susceptibility genes of type 1 AIH

	Susceptibility genes or alleles (protective alleles are underlined)	Populations	References
GWAS	DRB1*03:01, DRB1*04:01, *SH2B3*, *CARD10*	European	[34]
Candidate gene approach	DRB1*03:01, DRB1*04:01, DRB1*15:01	European	[35–38]
	DRB1*04:01, DRB1*04:05, DRB1*13:02, DRB1*15:01, DRB1*04/DRB1*08	Japanese	[39–44]
	DRB1*04:04, DRB1*04:05, DRB1*13:01, DRB1*13:02	Hispanic	[45–51]
	DRB1*04:05	Korean	[52]
	DRB1*01, DRB1*04, DRB1*08, DRB1*14	Indian, Iranian	[53–55]
	DRB1*13, DRB1*14	Pakistani	[56]
	DP5 (rs9277534G)	Japanese	[57]
	*KIR*	European	[58, 59]
	*PTPN22*	Japanese	[60]
	*SH2B3*	Japanese	[61]
	*TNFAIP3*	Japanese	[62]
	*STAT4*	Japanese	[63]
	*TNIP1*	Japanese	[64]
	*CTLA4*, *ICOS*	European, Japanese	[65–67]
	*FAS*	European, Japanese	[68, 69]
	*TNF*	European	[70, 71]

*AIH* autoimmune hepatitis, *GWAS* genome-wide association study

## *HLA* genes

A genome-wide association study (GWAS) is an association study of a genome-wide set of single nucleotide variants (SNVs) to find the contribution of common variants to susceptibility to disease. A GWAS of type 1 AIH was performed on European populations and a sole significant association for SNV in the *HLA* region was detected, indicating *HLA* as the strongest genetic risk factor for AIH [34]. In *HLA* association studies, DRB1*03:01 and DRB1*04:01 were associated with AIH in European populations [35]. It has been reported that DRB1*04:04, DRB1*04:05, and DRB1*13:01 are associated with AIH in Latin America [45–48]. DRB1*04:01 and DRB1*04:05 are associated with AIH in Japanese populations [39–42]. DRB1*08:02 and DRB1*08:03 also indicate predisposition to AIH in Japanese populations, when these alleles are possessed by individuals with DRB1*04:05 [42]. The associations of the *DRB1* heterozygous genotypes have also been reported for type 1 diabetes [72]. These associations in type 1 diabetes have been explained by the possible role of trans-complementing DQα-β heterodimer molecules, because of the strong linkage disequilibrium between *DR* and *DQ* loci. These molecules are composed of proteins encoded by the *DQA1* of one haplotype and the *DQB1* of the other. Analogically, the associations of the *DRB1* heterozygous genotypes in AIH can also be explained by the possible role of trans-complementing DQα-β heterodimer molecules. DRB1*04:05 has also been associated with AIH in Korean populations [52]. DRB1*01, DRB1*03, DRB1*04, DRB1*08, DRB1*13, and DRB1*14 have been associated with AIH in Indian and Iranian populations [53–55]. DRB1*13 and DRB1*14 have been associated with AIH in Pakistani populations [56]. Although AIH patients possessing DRB1*04 were predominantly female in European populations [73], this was not confirmed in our previous study [42].

DRB1*15:01 has been protectively associated with AIH in European and Japanese populations [35, 39]. DRB1*13:02 has also been seen to be protective against AIH in Latin America [46, 48, 49] and Japan [41]. However, DRB1*13:02 and DRB1*13:01 differ by only a single amino acid residue. DRB1*13:02 is known to be a common protective allele against several autoimmune diseases [74].

Type 2 AIH has been observed in European female children, but only a few studies have been conducted because of the low prevalence of the disease. DRB1*03 and DRB1*07 have been reported to be associated with risk of type 2 AIH [50, 75].

It has also been reported that an SNV in *DPB1*, rs9277534, is associated with AIH in Japanese populations [57]. The rs9277534G variant is known to be associated with the expression levels of *DPB1* [76, 77] and also shows strong linkage disequilibrium with DP5 alleles (DPB1*03:01, DPB1*05:01, DPB1*06:01, DPB1*09:01, DPB1*13:01, DPB1*14:01, DPB1*19:01, and DPB1*25:01) [57, 78, 79]. Similarly, the DP5 allele group was found to be associated with AIH in our analyses (Supplementary Table S1, unpublished), and DP5 tended to be associated with risk of AIH, when conditioned on *DRB1* alleles. On the other hand, DPB1*04:01 tended to be associated with the risk of AIH including cirrhosis (Supplementary Table S2, unpublished) and also exhibited this tendency of association when conditioned on *DRB1* alleles. These data suggest differing specificities of *DPB1* alleles between AIH per se and AIH with cirrhosis, confirming the heterogeneity of AIH.

## Non-*HLA* genes

The previous GWAS on European type 1 AIH also suggested associations of SNVs in *HLA*, *CARD10*, *SH2B3*, and *ICOS* [34]. The association between rs6000782 in *CARD10* and AIH was not observed in a replication study in Japanese populations [80]. CARD10 is a scaffold protein and plays a critical role in the activation of the nuclear factor-κB (NF-κB) pathway [81]. Although the reported SNV (rs3784504) is not polymorphic in Japanese populations, another SNV (rs11065904) in *SH2B3* was found to be associated with Japanese AIH [61]. SH2B3 is an adaptor protein regulating cytokine signaling and a negative regulator of T cell activation. This SNV may change the expression levels of the *SH2B3* gene. The reported SNV (rs3784504) in the GWAS is an SNV associated with celiac disease [82] and is also a protective factor against bacterial infection [83]. The SNV rs4325730, upstream of *ICOS*, was associated with AIH in a replication study in Japanese populations [65]. This SNV is in strong linkage disequilibrium with an SNV associated with celiac disease, rs4675374 [84, 85]. It has been reported that ICOS-deficient mice exhibit reduced germinal center formation [86]. Altered expression levels of ICOS molecules may change the development of autoreactive B cells via T follicular helper cells. Thus, some of the results from the GWAS have been confirmed in replication studies, and some risk SNVs of AIH were found to be common with those of celiac disease.

The candidate gene approach on type 1 AIH has been performed on several genes. Variants among the *CD28*, *CTLA4*, and *ICOS* gene clusters in 2q33.2 have been reported to be associated with autoimmune or inflammatory diseases. The products of *CD28*, *CTLA4*, and *ICOS* genes were CD28-family member molecules transducing co-stimulatory or inhibitory signals. An SNV (+49A/G) in the *CTLA4* gene is associated with AIH in European populations [66, 67], but this association was not confirmed in other studies [87, 88]. A meta-analysis of the SNV on AIH produced no significant associations [89]. An SNV (-670) upstream of the *FAS* gene is associated with AIH in Japanese populations [68] and is associated with AIH patients with cirrhosis at presentation in European populations [69]. The expression of Fas molecules on the cell surface leads to programmed cell death. The role of the *FAS* gene in AIH is supported by the results from animal models; *Fas*-deficient mice are less sensitive to Concanavalin A-induced hepatitis, an AIH animal model [90, 91]. An SNV (-308) in the *TNF* gene is associated with AIH in European populations [70, 71] and increases the serum levels of TNF-α, a proinflammatory cytokine [92]. An SNV (rs755622) in the promoter region of the *MIF* gene is associated with AIH severity in Japan and the USA [93]. MIF is a proinflammatory cytokine implicated in the pathogenesis of autoimmune diseases. An SNV (-590) in the promoter region of the *IL4* gene has been associated with pediatric AIH in Egypt [94]. IL-4 is a cytokine and induces a T helper 2 response. An SNV (rs7574865) in *STAT4* is associated with AIH in Japanese populations [63], and has been reported to be associated with various autoimmune diseases, including SLE [95]. STAT4 is a transcription factor activated by IL-12, leading to the activation of monocytes and to T helper 1 and T helper 17 cell differentiation; it is considered to play an important role in liver injury [96]. An SNV (rs2476601) in *PTPN22* is associated with various autoimmune diseases in European populations. This SNV is not polymorphic in Japanese populations. However, other SNVs (rs1217412, rs1217388, rs1217407, and rs2488458) in *PTPN22* are associated with Japanese AIH [60]. Lymphoid-specific protein tyrosine phosphatase is encoded by *PTPN22* and plays an important role in regulation of the activation of T cells.

An SNV (exon 2, restriction fragment length polymorphism with Fok I) in the *VDR* gene is associated with AIH in European and Chinese populations [97, 98]. Variants of the *VDR* gene are associated with various autoimmune diseases [99, 100]. Vitamin D receptor is encoded by the *VDR* gene and 1,25-dihydroxyvitamin D_3_, the active form of vitamin D, inhibits the production of cytokines by T cells [101]. An SNV (-1993) in the promoter region of the *TBX21* gene is associated with AIH in Chinese populations [102]. T-bet is encoded by the *TBX21* gene and is a regulator of the development of T helper 1 cells. SNVs (+ 869 and +915) in the exon 1 of the *TGFB1* gene are associated with AIH severity in Latin America [103]. These variants are associated with the production of TGF-β1, which causes tissue fibrosis.

*TNFAIP3* is a common susceptibility gene for autoimmune diseases, including SLE [104, 105]. A20 is encoded by the *TNFAIP3* gene and an inhibitor of the NF-κB signaling pathway. It has been reported that deleterious missense variants or loss of function variants in the *TNFAIP3* gene causes haploinsufficiency of A20 syndrome [106–108], an autoinflammatory disease with symptoms similar to those of Behçet’s disease. Deleterious missense variants change amino acid residues conserved across species. Deleterious SNVs in the exons of the *TNFAIP3* gene are associated with AIH patients with cirrhosis in Japanese populations [62]. In addition, the deleterious allele frequency of *TNFAIP3* increases in AIH patients without the *DRB1* risk alleles (DRB1*04:01, DRB1*04:05, DRB1*08:02, or DRB1*08:03) [42]. It has also been reported that A20 attenuates liver cirrhosis in nonalcoholic fatty liver disease [109], and that SNVs in *TNFAIP3* are associated with the severity of liver cirrhosis in patients with the human immunodeficiency virus and hepatitis C virus [110].

SNVs in the *TNIP1* gene are associated with SLE [111–113]. An adaptor protein binding to A20 is encoded by *TNIP1* and inhibits NF-κB activation by TNF-α. An SNV, rs7708392, in the *TNIP1* gene is associated with AIH in Japanese populations [64]. A stronger association is observed in AIH patients without the DRB1*04:05 allele, the strongest genetic risk factor for Japanese AIH.

Some HLA class I molecules are the ligands of the killer cell immunoglobulin-like receptors (KIRs) transducing inhibitory or activating signals. Age at onset of type 1 AIH is significantly associated with the *KIR2DS1* gene in European populations [58]. The increased frequency of the functional form of *KIR2DS4* in pediatric type 1 AIH has been detected and a synergistic effect observed in combination with DRB1*13:01 in Latin America [59].

Attempts have been made to explain the female predominance in autoimmune diseases by genetic factors: skewed X chromosome inactivation, X chromosome dosage, or microchimerism [114]. When X chromosome inactivation is skewed, X-linked antigens may escape presentation in the thymus. The increased incidence of autoimmune diseases has been observed in cases of Turner syndrome (45, X monosomy) and Klinefelter syndrome (47, XXY trisomy). Fetal stem cells enter the blood circulation of the mother during birth and microchimerism develops. Microchimeric cells may be targets as non-self cells. However, few studies have been published of these factors in AIH. There is only one single report on a Klinefelter syndrome patient with SLE and AIH [115].

## Conclusions and future perspectives

Although the influence of many genetic and environmental factors on the pathogenesis of AIH has been investigated, only some have been confirmed as risk factors for AIH. *DRB1* is the sole established genetic risk factor. Because the possession of DRB1*03 or DRB1*04 is included in the criteria of the International Autoimmune Hepatitis Group for diagnosis of type I AIH [17], the allele carrier frequencies of these alleles should be found to be greater in AIH patients. Although the effects may be limited, the influence of the risk *DRB1* alleles seems to have been overestimated. The effect of *DRB1* on the pathogenesis of AIH has been highlighted. However, *DRB1* and *DQB1* are in strong linkage disequilibrium and the role of *DQB1* in predisposition cannot be eliminated [42]. It is of interest that some SNVs in non-*HLA* genes are associated with AIH patients without the risk *DRB1* alleles [62, 64]. These data suggest that the weaker genetic risk factors in non-*HLA* genes cannot contribute to the predisposition to AIH of individuals with the other genetic risk factor when the gene–gene interaction is observed in AIH patients. Additionally, the clinical features of AIH patients with DRB1*04:05 differ from those without it [40, 42]. Thus, genetic analyses of AIH may provide an explanation for the heterogeneity of AIH.

Since type 1 AIH shares some clinical features with SLE [10–12], common susceptibility genes were to be expected between SLE and AIH. *HLA*, *STAT4*, *TNIP1*, *TNFAIP3*, and *PTPN22* were the common susceptibility genes detected between SLE and AIH. There were also some common predisposing genes detected between AIH and celiac disease: *HLA*, *SH2B3*, *ICOS*, *TNFAIP3*, and *STAT4* [82]. These data also suggest the existence of common signaling pathways in the pathogenesis of AIH and celiac disease. The associated alleles and the association manner of *HLA* in AIH were similar to those in type 1 diabetes, but not to those in rheumatoid arthritis [42]. Thus, some genetic factors of AIH seem to overlap with those of some autoimmune diseases.

The results from several GWASs should be reported, to estimate the similarity of the genetic factors. There has been only one GWAS on AIH [34], although many GWASs have been published on other diseases. In that one GWAS, significant associations of SNVs were found only in the *HLA* region. Some suggestive associations of SNVs with AIH were observed in non-*HLA* genes. Since AIH is a rare disease, it is quite difficult to enlarge the sample size of the GWAS. Because it is also a heterogeneous disease, a larger sample size is needed to detect the association of SNVs in non-*HLA* genes. Although many genes have been reported to be associated with AIH in different ethnic populations by the candidate gene approach, the GWAS could not confirm this. Different allele frequencies in different ethnic populations, the small sample size, the effects of various environmental factors, and the heterogeneity of the disease are the reasons for this result. A multi-ethnic, large-scale case-control study is needed to resolve the problem [116]. Such future studies on AIH are eagerly awaited, to shed light on its pathogenesis. The results of these GWASs should show a similarity between AIH and other autoimmune diseases [117]. However, a GWAS can detect the contributions only of common variants, not those of rare variants, structural variants, copy-number variants, gene families, gene–gene interactions, or gene–environment interactions. Some AIH patients present more complicated cases because of the accompaniment of cirrhosis, and their prognoses are worse [14]. SNVs specifically associated with AIH with cirrhosis have been detected (Supplementary Table S2) [62, 69] and may predict the prognosis of the patients. More than 80% of AIH patients respond to conventional treatments with corticosteroid, but less than 20% are refractory [13, 118]. Some genetic biomarkers for the prediction of the response to conventional treatments may be detected by analogy in the future, to establish a new strategy for the treatment of AIH.

The genetic analyses of AIH revealed the risk SNVs in *HLA* and non-*HLA* genes. Several susceptible genes have been confirmed so far. Although it is difficult to increase the sample size for this rare disease, larger-scale GWASs of different ethnic groups with precise clinical information are necessary, to confirm these susceptible genes and to clarify the gene–gene and gene–environment interactions. Additionally, genetic factors of AIH overlapped with those of other autoimmune diseases, suggesting an overlap of pathogenesis. Stratified analyses of AIH may clarify variations in the pathogenesis of disease subtypes and explain the obvious heterogeneity of AIH. In the near future, discrimination of treatment responders could be predicted by biomarkers generated from genetic analyses of AIH.

## Supplementary Information

**Additional file 1: Supplementary Table S1.** Logistic regression analysis of DPB1 alleles in AIH patients and controls.

**Additional file 2: Supplementary Table 2.** Logistic regression analysis of DPB1 alleles in AIH patients with cirrhosis and controls.

## Data Availability

All data are presented in the paper and in the supplementary material.

## Abbreviations

AIH: Autoimmune hepatitis
GWAS: Genome-wide association study
HLA: Human leukocyte antigen
IAIHG: International Autoimmune Hepatitis Group
KIRs: Killer cell immunoglobulin-like receptors
NF-κB: Nuclear factor-κB
SNVs: Single nucleotide variants
SLE: Systemic lupus erythematosus
