# Supplementary material for: Genetic risk factors for autoimmune hepatitis: implications for phenotypic heterogeneity and biomarkers for drug response
Source: Hum Genomics. 2021 Jan 28;15:6. doi: 10.1186/s40246-020-00301-4 (PMC7841991; doi:10.1186/s40246-020-00301-4)
Supplement: Supplementary file 1 — Additional file 1: Supplementary Table S1. Logistic regression analysis of DPB1 alleles in AIH patients and controls. [file 40246_2020_301_MOESM1_ESM.pdf]

Supplementary Table S1. Logistic regression analysis of *DPB1* alleles in AIH patients and controls.

	Unconditioned				Conditioned on *DRB1* alleles			
DPB1 allele	OR	95%CI	*P*	*P*c	$OR_{adjusted}$	95%CI	$P_{adjusted}$	$Pc_{adjusted}$
*DPB1*02:01*	0.77	(0.61–0.98)	0.0349	0.4884	0.78	(0.59–1.03)	0.0804	NS
*DPB1*02:02*	0.75	(0.44–1.27)	0.2848	NS	0.69	(0.38–1.27)	0.2353	NS
*DPB1*03:01*	1.19	(0.74–1.92)	0.4663	NS	1.12	(0.65–1.92)	0.6917	NS
*DPB1*04:01*	0.78	(0.49–1.24)	0.3028	NS	1.64	(0.77–3.52)	0.2026	NS
*DPB1*04:02*	1.07	(0.75–1.51)	0.7132	NS	0.98	(0.62–1.54)	0.9209	NS
*DPB1*05:01*	1.27	(1.04–1.56)	0.0192	0.2694	1.08	(0.85–1.38)	0.5168	NS
*DPB1*06:01*	1.54	(0.34–6.91)	0.5761	NS	2.25	(0.43–11.78)	0.3360	NS
*DPB1*09:01*	0.86	(0.61–1.20)	0.3716	NS	1.47	(0.77–2.82)	0.2472	NS
*DPB1*13:01*	0.47	(0.20–1.09)	0.0787	NS	0.58	(0.24–1.43)	0.2402	NS
*DPB1*14:01*	2.35	(1.00–5.57)	0.0512	0.7165	1.49	(0.54–4.09)	0.4372	NS
*DPB1*17:01*	0.76	(0.13–4.59)	0.7682	NS	1.83	(0.10–35.37)	0.6882	NS
*DPB1*19:01*	2.89	(0.56–15.01)	0.2057	NS	2.54	(0.42–15.45)	0.3116	NS
*DPB1*25:01*	1.15	(0.07–18.41)	0.9225	NS	3.67	(0.13–103.05)	0.4449	NS
*DPB1*41:01*	0.57	(0.05–6.34)	0.6493	NS	0.89	(0.06–14.16)	0.9315	NS
DP2	0.78	(0.63–0.95)	0.0128		0.82	(0.64–1.04)	0.1000	
DP5	1.29	(1.05–1.57)	0.0132		1.22	(0.96–1.55)	0.1091	

AIH: autoimmune hepatitis, OR: odds ratio, CI: confidence interval, *P*c: corrected *P* value, NS: not significant. Association was tested by logistic regression analysis. Association was tested by logistic regression analysis. $P_{adjusted}$ and $OR_{adjusted}$ were calculated by conditional logistic regression analysis under the additive model. Corrected *P* (*P*c) values were calculated by multiplying the *P* value by the number of alleles tested. Allele group DP5 includes *DPB1*03:01, *05:01, *06:01, *09:01, *13:01, *14:01, *19:01,* and **25:01* and allele group DP2 includes *DPB1*02:01, *02:02, *04:01, *04:02, *17:01,* and **41:01*.
