# Supplementary material for: Genetic risk factors for autoimmune hepatitis: implications for phenotypic heterogeneity and biomarkers for drug response
Source: Hum Genomics. 2021 Jan 28;15:6. doi: 10.1186/s40246-020-00301-4 (PMC7841991; doi:10.1186/s40246-020-00301-4)
Supplement: Supplementary file 2 — Additional file 2: Supplementary Table 2. Logistic regression analysis of DPB1 alleles in AIH patients with cirrhosis and controls. [file 40246_2020_301_MOESM2_ESM.pdf]

Supplementary Table 2. Logistic regression analysis of *DPB1* alleles in AIH patients with cirrhosis and controls.

	Unconditioned				Conditioned on *DRB1* alleles			
DPB1 allele	OR	95%CI	*P*	*P*c	$OR_{adjusted}$	95%CI	$P_{adjusted}$	$Pc_{adjusted}$
*DPB1*02:01*	0.81	(0.46–1.40)	0.4454	NS	1.03	(0.56–1.91)	0.9176	NS
*DPB1*02:02*	0.86	(0.26–2.86)	0.8111	NS	1.19	(0.30–4.74)	0.8076	NS
*DPB1*03:01*	1.69	(0.69–4.14)	0.2526	NS	1.49	(0.54–4.11)	0.4455	NS
*DPB1*04:01*	2.04	(0.98–4.24)	0.0574	0.5165	5.60	(1.13–27.63)	0.0346	0.2768
*DPB1*04:02*	1.46	(0.73–2.90)	0.2843	NS	1.50	(0.65–3.46)	0.3404	NS
*DPB1*05:01*	0.89	(0.57–1.40)	0.6135	NS	0.70	(0.41–1.21)	0.2018	NS
*DPB1*06:01*	3.33	(0.34–32.78)	0.3019	NS	5.27	(0.32–85.68)	0.2432	NS
*DPB1*09:01*	0.77	(0.35–1.73)	0.5299	NS	1.09	(0.25–4.72)	0.9096	NS
*DPB1*14:01*	1.23	(0.15–10.12)	0.8442	NS	NA	NA	NA	NA
DP2	1.17	(0.75–1.81)	0.4877		1.57	(0.90–2.73)	0.1091	
DP5	0.86	(0.55–1.33)	0.5001		0.64	(0.37–1.11)	0.1141	

AIH: autoimmune hepatitis, OR: odds ratio, CI: confidence interval, *P*c: corrected *P* value, NS: not significant, NA not applicable. Association was tested by logistic regression analysis. $P_{adjusted}$ and $OR_{adjusted}$ were calculated by conditional logistic regression analysis under the additive model. Corrected *P* (*P*c) values were calculated by multiplying the *P* value by the number of alleles tested. Allele group DP5 includes *DPB1*03:01, *05:01, *06:01, *09:01, *13:01, *14:01, *19:01,* and **25:01* and allele group DP2 includes *DPB1*02:01, *02:02, *04:01, *04:02, *17:01,* and **41:01*.
